# Effect of Executive Function on Event-Based Prospective Memory for Different Forms of Learning Disabilities

**DOI:** 10.3389/fpsyg.2021.528883

**Published:** 2021-03-05

**Authors:** Lili Ji, Qi Zhao, Huang Gu, Yanan Chen, Junfeng Zhao, Xiaowei Jiang, Lina Wu

**Affiliations:** ^1^Institute of Cognition, Brain and Health, Henan University, Kaifeng, China; ^2^Institute of Psychology and Behavior, Henan University, Kaifeng, China; ^3^Department of Psychology, University of Macau, Macau, China

**Keywords:** shifting, inhibition, updating, learning disabilities (LDs), event-based prospective memory, executive function

## Abstract

Students with learning disabilities (LDs) suffer from executive function deficits and impaired prospective memory (PM). Yet the specificity of deficits associated with different types of LDs is still unclear. The object of the present research was to compare subgroups of students with different forms of LDs (<25th percentile) on executive function and PM. Students with a mathematics disability (MD, *n* = 30), reading disability (RD, *n* = 27), both (RDMD, *n* = 27), or neither (typically developing, TD, *n* = 30) were evaluated on a set of executive functioning tasks (e.g., updating, inhibition, and shifting) and on PM. The results showed that students with MDs and RDMDs suffered from PM deficits. Among the subtypes of LDs, the deficit is different. The students with RDMDs showed a wide range of defects in PM, shifting, inhibition, and updating. In comparison, students with MDs experienced deficits in PM and shifting, while students with RDs experienced a deficit only in updating. For the RD group, the RDMD group and the TD group, updating, and shifting significantly predicted PM. For the MD group, only shifting significantly predicted PM performance, but PM deficits were not completely confined to shifting deficits.

## Introduction

Prospective memory (PM) refers to the memory of expected behavior to be performed at an appropriate time or situation in the future. There are two ways in which PM retrieval occurs: event-based PM is when an environmental cue signals the appropriate time to perform an intended action, such as remembering to do one’s homework when seeing one’s school bag; a time-based PM, such as remembering to go to a meeting at 16:00. Due to the presence of external cues, event-based PM tasks are simpler than time-based PM tasks, which require more self-initiation (Williams et al., 2013; Li et al., 2014). PM plays an important role in people’s daily cognition and daily life activities.

Prospective memory tasks are common in daily life, and PM errors may account for more than half of all daily memory problems (Crovitz and Daniel, 1984). Consistent with the universal nature of PM, the failure of PM will hinder students’ autonomy and independence. Therefore, the influence of cognitive mechanism on the performance of PM has attracted more and more attention (Einstein and McDaniel, 1996; Berg et al., 2004; Rendell et al., 2009; Schnitzspahn et al., 2013; Mahy et al., 2014; Spiess et al., 2015; Chen et al., 2017). For example, problems such as remembering to do their homework or to prepare and review lessons may increase the need for external assistance to prevent those everyday PM failures.

Several studies have shown that PM is significantly related to learning disabilities (LDs). Students with LDs, especially LDs in math (MD) report more PM failures (Dong et al., 2008; Chen et al., 2017, 2018). Dong et al. (2008) compared the effects of cognitive style and reminders on event-based, time-based, and activity-based PM tasks between LDs and typical developing students. The results showed that the performance of students with LDs was not as good as that of typical students in time-based PM tasks without reminders and that the reminders could facilitate the performance of students with LDs in time-based PM tasks but did not improve their performance in event-based PM tasks. Chen et al. (2017) studied the effects of the working memory (WM) demand and reminders for an event-based PM task between students with LDs (below the 20th percentile) and high scores (above the 20th percentile) in math. The results indicated that high-scoring students performed better than the LD students in all PM and N-back tasks. The reminder improved PM performance and thus reduced prospective interference and a high WM load had more influence on LD students than on high-scoring students. The results suggested that students with LDs in math had poor PM and were affected more by the high WM load. Chen et al. (2018) explored the effects of target salience and task importance on PM, and prospective interference was compared between LD students and high-scoring pupils in math. The results suggested that high-scoring pupils outperformed LD students in PM tasks, and salient targets improved PM performance.

However, our knowledge about the influencing factors and cognitive mechanisms of PM in LDs is still limited. A previous study defined LDs based on lower-order skills, calculations for math disability (MD) and word reading for reading disability (RD), which are the dominant classification strategies in the LD learning disabilities literature. RDs and MDs are common and often occur together (RDMD), and they are typically defined by reading and math subtests of the same battery of standardized achievement tests (e.g., Barbaresi et al., 2005; Landerl and Moll, 2010; American Psychiatric and Association, 2013). Few studies have extended to all subtypes of LDs, and those conducted have mainly focused on RDs or MDs. Therefore, knowledge on students’ outcomes related to comorbid conditions (RDMDs) or the comparison of RDs and MDs is limited. To begin to address this gap in the literature, the current study aimed to explore the effect of the cognitive mechanism on PM performance in students with RDs only, MDs only, RDMDs, and students with neither disorder.

### Effect of Executive Function on Prospective Memory

Executive function is a high-level cognitive process of students and the core of students’ cognitive function which can make students produce purposeful and coordinated behaviors, including three functional domains: shifting, updating, and inhibition (Miyake et al., 2000; Mackinlay et al., 2006; Mäntylä et al., 2006; Sluis et al., 2007; Altgassen et al., 2009; Li et al., 2014). Impairments in these aspects of executive functioning may be relevant for failure of PM performance (Mäntylä, 2003). PM requires the integration of several complex processes, which is remembering to complete an intention at the appropriate time (McDaniel and Einstein, 2007; Williams et al., 2013; Li et al., 2014) and consists of several complex processes. First of all, one must plan future actions, and processing other information while maintaining the intention to complete the action. Second, when either an event-based or a time-based cue is presented, one has to retrieve the PM, inhibit and flexibly switch from an ongoing task to the planned action (Kliegel et al., 2002). Therefore, successful PM requires a certain level of executive functioning ability (Miyake et al., 2000; Mackinlay et al., 2006; Altgassen et al., 2009; Azizuddin, 2014; Yang et al., 2019).

Executive functions are assumed to be involved in PM (Burgess et al., 2001; Smith-Spark et al., 2016). A large number of studies have indicated that executive function plays an important role in PM (Kvavilashvili et al., 2001; Ward et al., 2005; West, 2007; Wang L. et al., 2008; Mackinlay et al., 2009; Rendell et al., 2009). The three executive functions used in this study, shifting, inhibition, and updating, are based on Baddeley and Della Sala’s (1996)’s specified three component functions. The three functions have proved to be correlated but partially independent cognitive constructs, and they are related functions that share some potential commonality, common mechanisms spanning different executive functions or functions putatively performed by the frontal brain areas (Miyake et al., 2000; Ross et al., 2016). Updating abilities maintain the intention of PM (Mahy and Moses, 2011; Mahy et al., 2014). Shifting is mainly reflected in the ability to involve shifting between two tasks (ongoing and PM tasks) when PM cues appeared to be a key factor in the successful implementation of PM (Spiess et al., 2015). Similarly, inhibition is necessary when a habitual dominant action must be inhibited and replaced by a new one (Berg et al., 2004).

Despite the repeatedly expressed idea that EF and PM may be related, only a few studies so far have explored the role of distinct EF facets in PM performance (Schnitzspahn et al., 2013). In addition, the accumulated evidence showed that the three components of executive function were not able to consistently predict PM (Mackinlay et al., 2009; Ford et al., 2012; Schnitzspahn et al., 2013). Mackinlay et al. (2009) found that students with higher shifting had better PM performance, and updating had no significant influence on event-related PM. Ford et al. (2012) indicated that inhibition control rather than updating can predict PM in children aged 4–6 years. Schnitzspahn et al. (2013) investigated the role of three main executive functions (i.e., shifting, updating, and inhibition) in PM in young and older adults, and the results showed that inhibition and shifting were strong predictors of PM performance, which also explained the age difference of PM performance. However, there was no correlation between updating and PM performance in adulthood. In other words, the existing bounded studies have discussed the relationship between event-related PM and executive function, but the results of the previous studies were inconsistent regarding the predictive effect of various components of executive function on PM.

### Defects of Executive Function in Learning Disabilities

A substantial number of studies showed students with LDs have problems in executive function (e.g., McLean and Hitch, 1999; Hooper et al., 2002; Sluis et al., 2004; Swanson et al., 2006; Locascio et al., 2010). The current research explored the effect of subtypes of LD on the specificity of deficits in executive functions.

Studies have suggested that students with RDs perform poorly in reading comprehension, problem solving, and updating tasks than typical students (Cornoldi et al., 2012), especially in word updating tasks (Pelegrina et al., 2015). Students with MDs performed as well as normal students in word updating tasks, but they performed poorly in number updating tasks (Iuculano et al., 2011). Additionally, some studies have found that students with MDs displayed shifting and inhibition deficits (Jiao, 2014; Jiao and Liu, 2018). Compared to typical students, RD students performed worse in the inhibitory control and attention switching tasks (Hari and Renvall, 2001; Leong et al., 2007). Students with comorbid disabilities were generally deficient in WM updating, inhibition, and shifting of executive function (Sluis et al., 2004; Wang et al., 2008a; Peng et al., 2013; Zhang et al., 2016; Deng et al., 2018). Based on previous findings, it was found that different types of LDs may have heterogeneous cognitive defects heterogeneity (Andersson, 2010; Peng and Fuchs, 2014).

Compared with those of typical students, the defects of students with LDs in executive function (WM or inhibitory control or shifting) may be one of the most notable reasons for their lower learning potential (Söderqvist et al., 2011). Evidence from the nervous system offers more compelling proof. Brain activity related to executive function exhibits abnormal patterns in students with LDs (Gu et al., 2019), meaning that their ability to monitor information is less developed versus that of typical students, and this ability might be impeded when they learn new knowledge and invoke old knowledge that may reduce their learning efficiency.

### The Current Study

In the current study we focused on disentangling the significance of the three facets of executive control (shifting, inhibition, and updating) for predicting PM performance among students with different types of LDs (groups with RD only, MD only, RDMD, and a control group with neither disorder) who completed an extensive battery of measures of PM and executive functions. The specific predictions were as follows:

(1) Based on the study of executive function defects in LDs, we tentatively predicted that weaknesses in updating, inhibition, and shifting would emerge as significant weaknesses in groups with RDMD. In contrast, we expected updating difficulties to be associated with RDs but not MD, and anticipated that the group with MDs would exhibit a specific deficit in shifting.
(2) Based on the RDs, MDs, and RDMDs that define the groups, we anticipated that the PM deficits would be most pronounced in the RDMD group. We also hypothesized that the PM level of students with LDs would be lower than that of typical students. As previous research did not distinctly separate RDs and MDs, and the existing literature on RDs and PM is rather scarce, no specific prediction was formed in this regard.
(3) Regarding to the effect of executive function deficits on predicting PM deficits in students with different forms of learning difficulties, we expected students with MDs to show poor performance on measures and that the ability of shifting would significantly predicted PM performance. However, for the RD group, we expected that updating would significantly predict PM performance.

## Materials and Methods

### Participants

Twenty-seven students with RDs (15 male, age: 13.59 ± 0.50 years), 30 students with MDs (18 male, age: 13.46 ± 0.57 years), 27 students with RDMDs (13 male, age: 13.59 ± 0.64 years), and 30 TD students (16 male, age: 13.23 ± 0.67 years) were recruited from a junior middle school in Henan (grades: 7–9). All students were enrolled in regular classrooms. Before enrolling in the study, they were excluded for left-handedness, color-blindness, and possible mental disorders.

The inclusion eligibility criteria for the LD group were as follows (Wang and Liu, 2007; Zhang et al., 2016; Xiang et al., 2018): (1) students who had no problems in vision, hearing, motor skills, emotion, social and cultural adjustment, etc., were recruited; (2) all 1,102 students who participated in the experiment were tested with the Learning Adaptability Test (AAT) (Zhou, 1992); (3) the 172 students whose AAT scores were converted to grades 2 or below were invited to participate in the following PRS test; (4) the head teachers of these students filled out an adapted Chinese version of the pupil Rating Scale (PRS) (Zhou and Li, 2002), including 24 items rated on a 5-point scale ranging from 1 to 5, to characterize the students’ academic difficulty, and screened the 93 students who received scores less than 65 as suspected LDs; (5) the average of scores that these 93 students received on the latest two academic exams were transformed into *Z* scores. Some studies have suggested that students with Reading (language) scores below the 25th percentile and Mathematics scores above average were considered as RDs; those with Mathematics scores below the 25th percentile and Reading scores above average were considered as MDs; and students with both (Mathematics and Reading) scores below the 25th percentile were considered as students with RDMDs (Wang and Liu, 2007; Xiang et al., 2018; Gu et al., 2019). These criteria were applied to 27 students in the given RDs sample, 30 students in the given MD sample and 28 students in the given RDMD sample. Ultimately, one low- IQ student was excluded with the use of the Raven Standard Progressive Matrices (RSPM) (Zhang, 1985), and 84 students with LDs were included.

All the typically developing participants in the control group need to meet the following criteria: (1) the scores of the AAT and scores of two academic exams are above the middle level; (2) the scores of the PRS are more than 65; (3) the RSPM score is at the normal level; (4) matched on intelligence.

The demographic differences of the four groups were listed in Table 1. The effect for ages, *F*_(3,110)_ = 1.90, *p* = 0.13 and IQ, *F*_(3,110)_ = 2.07, *p* = 0.11, showed no significant differences between all the groups. With respect to the score of Learning Adaptability Test and Pupil Rating Scale, there was no significant difference between MD group and RD group, and TD groups outperformed the MD and RD group. RDMD group showed significantly poorer performance than the other three groups. In terms of Reading score, there was no significant difference between MD group and TD group, and both groups showed significantly better performance than the RD and RDMD group. With respect to Mathematics score, there was significant difference between RD group and TD group, and both groups outperformed the MD and RDMD group.

**TABLE 1:** Means and standard deviations and demographic characteristics and group effects for test scores for the four groups.

Construct	RD	MD	RDMD	TD	*Post hoc* (Bonferroni)
*N*	27	30	27	30	
Age(year)	13.59 (0.50)	13.46 (0.57)	13.59 (0.64)	13.23 (0.67)	TD = RD = MD = RDMD
Gender(M:F)	15:12	18:12	13:14	16:14	
AAT	42.59 (1.62)	41.30 (2.22)	34.71 (3.77)	55.26 (3.01)	TD > RD = MD > RDMD
PRS	56.11 (1.62)	55.83 (2.52)	43.71 (5.99)	67.32 (3.96)	TD > RD = MD > RDMD
RS	57.90 (10.11)	68.19 (4.27)	48.80 (9.71)	73.44 (5.32)	TD = MD > RD > RDMD
MS	66.07 (8.38)	37.22 (6.58)	28.91 (10.55)	78.68 (3.82)	TD > RD > MD > RDMD
RSPM(IQ)	48.76 (3.16)	48.03 (2.97)	47.86 (2.61)	49.55 (2.94)	TD = RD = MD = RDMD

*RD, reading disability; MD, mathematics disability; RDMD, reading and mathematics disabilities; TD, typically developing; AAT, Learning Adaptability Test; PRS, Pupil Rating Scale; RS, Reading score; MS, Mathematics score; RSPM, Intelligence score.*

### Materials and Procedure

The students used a laptop computer equipped with Tool book and E-prime2.0 software for individual testing. The experiments were conducted in a quiet, sound attenuated room, and subjects were seated with their eyes approximately 100 cm from a 17-in screen. All stimuli were displayed in the center of the screen (4.0 × 4.6 visual angle). Each participant took part in two 40-min sessions 1 week apart. In session 1, participants were required to complete the PM task, the Stroop color-word test. In session 2, they were presented with the N-back task, the More-odd shifting task (MOS). The order of the tests was counterbalanced for sessions. Analyses indicated no effect of presentation order on performance of any of these tests.

### Executive Function Tasks

The role of executive function in students’ PM performance was examined with several tasks: The N-back task, the Stroop color-word test, and the MOS.

#### Updating

Updating was measured using the N-back task. In the task, a series of one-digit numbers (from 1 to 9) in a random sequence was shown to the participants in the center of the computer screen (4.0° × 4.6° visual angle). Task difficulty was varied using three workloads (0-back, 1-back, 2-back). Participants were asked to press the “F” button with one index finger when the number that appeared on the screen was the same as the target number 3 for the 0-back task, the number presented one trial back for the 1-back task and the number presented two trials back for the 2-back task. Conversely, the participants were asked to respond by pressing the “J” button if the presented number did not meet the “match” criterion (Yuan et al., 2016). The match/non-match buttons were counterbalanced for the left/right hand across the participants. The duration of the stimulation was 300 ms, and the interval of stimulation (ISI) was 1,600 ms. Each type of N-back task contained 100 stimulus trials presented in pseudo-random order, with 50 match trials and 50 non-match trials. The stimulus at each workload level was divided into five blocks, and each block contained 20 trials. The correct response time and accuracy to the target stimuli were collected.

#### Inhibition

Inhibitory control was measured using the Stroop color-word test (Hammes, 1973). In the Stroop color-word test (SCWT), the experimental materials were the three words “红” (meaning red), “绿” (meaning green), “蓝” (meaning blue.), which were presented in three different font colors (red, green or blue). The whole task contained 120 stimulus trials including 60 congruent stimulus (the color of “绿” was green) and 60 incongruent stimulus (the color of “绿” was red). Each trial began with a fixation “+” for 500 ms, followed by a blank screen lasting for a random duration of 300–500 ms. After the blank screen, subjects saw a color word on the screen for 1,000 ms and the participants were instructed to identify the color of the Chinese characters by pressing the appropriate buttons (“7,” “8,” or “9” on the keyboard) corresponding to “red” (right index finger), “green” (right middle finger) or “blue” (right ring finger), respectively, without considering their meaning. If they did not respond within 1,000 ms, the picture would disappear and their response would be coded as missing. If subjects gave a response within 1,000 ms, the next trial would begin after an interval of 800–1,000 ms. Prior to the actual experiment, we conducted a training session to ensure that participants fully understood the task. Inhibitory control on the Stroop test was calculated as the difference between incongruent reaction time and congruent reaction times.

#### Shifting

To assess shifting, we used the well-established MOS referring to Salthouse’s paradigm (Salthouse et al., 2003). In the MOS, a series of numbers (1–4 and 6–9) were displayed on the center of the screen; each number appeared for 2,000 ms, and the stimulus interval (ISI) was 300 ms. The participants were asked to judge the number (1–9), not including 5. There were three conditions of judgment requirements in the test: (1) if the number appearing on the screen was greater than 5, participants were required to press “A” as quickly as possible. and if the number appearing on the screen was less than 5, participants were required to press “L” as quickly as possible; (2) if the number appearing on the screen was odd, participants were required to press “A” as quickly as possible, and if the number appearing on the screen was even, participants were required to press “L” as quickly as possible; and (3) when the number was colored black, participants were required to press “A” as quickly as possible if the number presented on the screen was more than 5 and “L” if it was less than 5; when the color of the number was blue, participants were required to press “A” or “L” after judging the parity of the number. The stimulus under each judgment condition was divided into two blocks (a, a, b, b, c, c). In the one shifting block (c), consisting of 48 trials, participants regularly alternated between the two conditions, switching from one to the other on every two trials. Therefore, the shifting block included 23 switch trials and 25 non-switch trials. The non-shifting blocks (a or b) included 24 trials of one condition and did not need a switch (all trials were non-switch trials). Participants were required to finish two shifting blocks and four non-shifting blocks (two blocks of each condition) in the order “abccba.” The participants were instructed to practice 8 trials before control block a or b and practice 16 trials before shifting block c. The switch cost was the difference between the average RTs of the switch trials in the shifting blocks and the average RTs of non-switch trials in the non-shifting blocks.

### Prospective Memory (PM) Tasks

In the study we adopted dual task paradigm used in the study of West et al.’s (2003). The ongoing task involved a color discrimination task of phrases. On ongoing activity trials, two colored phrases were presented in the center of a computer screen. The participants were asked to judge whether two phrases were of the same color. In the PM task, participants were required to pause the ongoing task and switch to the PM task by pressing the appropriate button when the same two phrases in coding stages PM target cues, which appeared on the screen again.

The phrases were obtained on the basis of category naming experiments. In the category naming experiment, 27 categories familiar to the subjects were selected (Posnansky, 1978), and the subjects were asked to list 8 examples for each given category. The samples listed in the first 12 digits of the cumulative frequency were randomly selected from each category to form 648 pairs, which were divided into two groups of 324 pairs each. Half of the word pairs were of the same color, for example “Word1–Word2 (both words presented in red)”, the other half were of different colors, for example “Word1 (presented in green) – Word2 (presented in blue). We use e-prime2.0 programming to ensure a rigorous experimental procedure.

There were 27 blocks each of which consisted of three stages. In the coding stage: two PM cue trials (each one consisting of two phrases with the same semantics and color) were presented for 2,000 ms followed by 1,000 ms of blank screen. Participants were asked to remember the two phrases, including their semantics and colors (PM memory tasks). In the distraction task stage: a three-digit number appeared on the screen after the coding stage lasting 3,000 ms, and participants were required to perform a “minus three” task to avoid repeating items they had just learned. In the ongoing task stage each block contained 24 trials which were divided into 22 ongoing activity trials and 2 PM cues (PM tasks were embedded in the ongoing tasks). Participants would press the “1” key if they judged that the colors of the two phrases were same and the “2” key if the colors of the two phrases were different without considering the semantics. However, participants were instructed to press the “0” key if they saw the previous PM target cues again as presented in coding stages on the screen without making relevant judgments. In each block, PM target cues occurred once in the first 11 trials and once in the last 11 trials. The correct response time and accuracy to the PM target cues were calculated as the dependent measure. Before the formal experiment, participants were allowed to complete a practice block to ensure that they fully understood the whole experimental procedure.

### Data Analysis

One-way multivariate analyses of variance (MANOVAs), one-way analyses of variance (ANOVAs), and Cohen’s *d* effect size (η_p_^2^) were calculated to determine differences between groups on each task. Bonferroni correction was applied for all analyses when multiple comparisons were made. Correlation analysis, stepwise regression analysis and hierarchical regression-based analysis were used to further determine whether executive function could explain the differences between the TD group and each of LD groups on PM tasks.

## Results

SPSS 20.0 was conducted for data collection and statistical analysis. The reaction time data of each participant for false responses and non-responses were deleted. RTs data was calculated based on a cutoff value of ±3 SD from the mean per participant (Chen and Du, 2017). Given the floor effect in 0-back and ceiling effect in 2-back task for about 13 years old of LD, respectively, only the accuracy and response time of the 1-back condition were used as the measure in this study.

**FIGURE 1:**
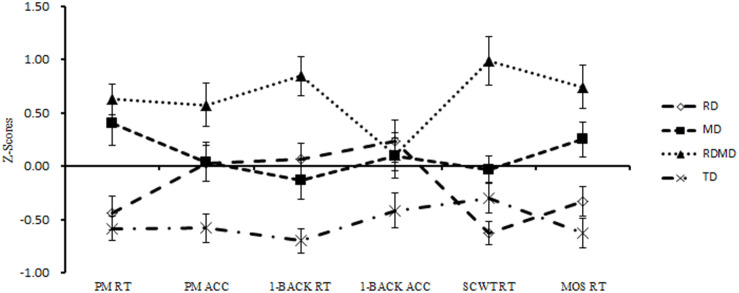
*Z*-scores on executive function tasks and PM by LD subgroups. The *Z*-scores for acc-related tasks (PM and 1-back) are reversed. PM, prospective memory; 1-back, N-back task (updating); SCWT, Stroop color-word test (inhibition); MOS, more-odd task (shifting); ACC, accuracy; RT, response time.

Descriptive statistics for the executive functioning tasks, PM task of each group and comparison of performances for all the groups are displayed in Table 2. To make the performance on the different task comparable, we transformed the performance on the different tasks into *Z*-scores by standardization based on all the participants. Figure 1 presented the performances of groups on different tasks.

**TABLE 2:** Comparison of performances on the PM task and N-back, SCWT, and More-odd shifting tasks for the four groups.

	RD (*n* = 27)	MD (*n* = 30)	RDMD (*n* = 27)	TD (*n* = 30)	*F*_(3,110)_	*p*
PM ACC(%)	0.67 (0.19)	0.67 (0.22)	0.54 (0.23)	0.80 (0.16)	7.45	<0.001
PM RT(ms)	956.96 (135.25)	1,094.37 (185.38)	1130.56 (122.71)	933.68 (102.9)	13.86	<0.001
1-back ACC(%)	0.73 (0.15)	0.74 (0.17)	0.74 (0.12)	0.82 (0.13)	2.52	0.07
1-back RT(ms)	160.51 (38.91)	151.03 (48.66)	199.10 (46.41)	122.96 (30.80)	16.08	<0.001
SCWT RT(ms)	46.61 (2.90)	49.69 (3.59)	54.93 (5.99)	48.28 (3.86)	19.76	<0.001
MOS RT(ms)	302.88 (45.13)	339.33 (54.81)	370.37 (67.39)	284.26 (46.59)	14.22	<0.001

*PM, prospective memory; 1-back, N-back task (updating); SCWT, Stroop color-word test (inhibition); MOS, more-odd task (shifting); ACC, accuracy; RT, response time.*

### Group Differences on Executive Functioning Tasks

Firstly, a one-way MANOVA was conducted on RTs and accuracy of N-back, RTs of SCWT and MOSs and revealed a significant medium group main effect, Wilks’s *k* = 0.47, *F*_(9,263)_ = 10.83, *p* < 0.001, η_p_^2^ = 0.23. Secondly, ANOVAs were run for N-back, SCWT, and MOSs, respectively.

#### N-Back Task: Updating

For the mean RTs for the N-back task, there was a main effect of group condition, *F*_(3,110)_ = 16.08, *p* < 0.001, η_p_^2^ = 0.31; Further pairwise comparisons with Bonferroni adjustment revealed that the TD group, RD group, and MD group performed significantly better than the RDMD group (*ps* < 0.001). The TD group also performed significantly better than the RD group (*p* < 0.01). The TD group outperformed the MD group at a marginally significant level (*p* = 0.06), and the RD versus MD contrast was not significant (*p* = 0.39). The same analysis on N-back accuracy revealed that, there was no significant main effect of group.

#### Stroop Color-Word Test: Inhibition

The ANOVA revealed significant large group differences on the mean RTs of the SCWT task, *F*_(3,110)_ = 19.76, *p* < 0.001, η_p_^2^ = 0.35; Further pairwise comparisons with Bonferroni adjustment revealed the RTs of the TD group, RD group and MD group were significantly shorter than the RTs of the RDMD group (*ps* < 0.001) and RD group was significantly shorter than the RTs of the MD group (*p* < 0.05), and there were no significant differences between the TD and RD groups and between the TD and MD groups (*ps* > 0.01).

#### More-Odd Shifting Task: Shifting

For the mean RTs of the MOS task there was a main effect of group, *F*_(3,110)_ = 14.22, *p* < 0.001, η_p_^2^ = 0.28; Further pairwise comparisons with Bonferroni adjustment revealed the RTs of the TD group was significantly shorter than the RTs of the MD group and RDMD group (*ps* < 0.001). RD group was significantly shorter than the RTs of the RDMD group (*p* < 0.001), but there was no significant difference between the other two groups (*ps* > 0.01).

### Group Differences on Prospective Memory Task

Similarly, a one-way MANOVA was conducted on RTs and accuracy of the RTs and accuracy of PM task and revealed a significant medium group main effect, Wilks’ λ = 0.64, *F*_(6,218)_ = 9.28, *p* < 0.001, η_p_^2^ = 0.20.

Two ANOVAs were run for the RTs and accuracy of PM task, respectively. For the mean RTs of the PM task there was a main effect of group, *F*_(3,110)_ = 13.86, *p* < 0.001, η_p_^2^ = 0.27; Further pairwise comparisons with Bonferroni adjustment revealed that the RTs of the TD group and RD were significantly shorter than the RTs of the MD group and RDMD group (*ps* < 0.001), but there were no significant difference between the TD and RD groups (*p* = 0.53) and between the MD and RDMD groups (*p* = 0.33). The same analysis on PM accuracy showed that, there was a main effect of group, *F*_(3,110)_ = 7.45, *p* < 0.001, η_p_^2^ = 0.17. Further analysis showed that performance was best in the TD group, which was significantly higher than the RDMD group (*p* < 0.001), but there was no significant difference between the other two groups (*ps* > 0.01).

### Correlation Analysis Between Prospective Memory and Executive Function

Pearson’s correlation (see Table 3) was used in analyzing the relation of PM tasks and executive functioning tasks performance for RD, MD, RDMD, and TD groups respectively. Results showed that PM response time for all the four groups was significantly positive correlated with the response time of updating (*ps* < 0.05) and shifting (*ps* < 0.05). However, PM response time was not correlated with the response time of inhibitory control for all the four groups.

**TABLE 3:** Correlation between the average RTs of PM and three components of executive function.

		PM	1-back	SCWT	MOS
	RD		0.53**	0.34	0.47*
PM	MD		0.37**	0.07	0.38**
	RDMD		0.64***	0.29	0.71***
	TD		0.70***	0.37	0.58***
1-back	RD			0.30	0.23*
	MD			0.36*	0.33
	RDMD			0.30	0.51**
	TD			0.20	0.47**
SCWT	RD				0.21
	MD				0.26*
	RDMD				0.36*
	TD				0.37*
MOS	RD				
	MD				
	RDMD				
	TD				

*p < 0.05; **p < 0.01; ***p < 0.001.

### Regression Analysis Between Prospective Memory and Executive Function

Stepwise multiple regressions were performed to examine the impact of updating, inhibitory control and shifting on PM response time respectively. For the RD group, updating and shifting significantly predicted the PM and explained 36.4% the variance in the PM. For the MD group, shifting significantly predicted the PM and explained 11.6% the variance in the PM. For the RDMD group, updating and shifting significantly predicted the PM and explained 56.8% the variance in the PM. For the TD group, updating and shifting significantly predicted the PM and explained 53.3% the variance in the PM. Table 4 presents the significant predictors of the models.

**TABLE 4:** Regressions models predicting students’ performance in the prospective memory (PM) task.

**Group**	**Variable**	***T***	***p***	***R*^2^**	***β***
RD	1-back	2.31^∗^	0.030*	0.36	0.37
	MOS	2.76^∗∗^	0.009**		0.46
	SCWT	0.96	0.371		0.15
MD	MOS	2.19^∗^	0.037*	0.12	0.38
	1-back	0.54	0.162		0.28
	SCWT	0.08	0.362		0.14
RDMD	1-back	2.50^∗∗^	0.008**	0.57	0.38
	MOS	3.38^∗∗^	0.003**		0.11
	SCWT	0.28	0.661		0.24
TD	1-back	3.81^∗∗^	0.001**	0.53	0.32
	MOS	2.19^∗^	0.037*		0.55
	SCWT	0.97	0.341		0.13

*p < 0.05; **p < 0.01; ***p < 0.001.

Furthermore, a regression-based analysis method was conducted to further examine whether the executive function deficits explain the PM difference between the TD group and other three groups. By using the dummy coding, we created the variable “group” was dummy-coded with TD as reference group (Cohen et al., 2003). The effect of executive function within each aspects on PM was controlled by putting N-back, SCWT and MOS tasks in the model 1 of PM tasks (see Table 5). As presented in Table 5, Models 1 and 2 showed that after controlling for the effect of executive function, there was no significant group effect between the TD and RD groups and between the TD and RDMD groups on PM tasks, and also no significant interaction effect between the group and executive functions (*ps* > 0.05). Only updating and shifting still significantly predicted the PM (*ps* < 0.05), and the MD groups still performed poorer than the TD group on PM tasks. Thus, PM deficits in students with MD groups cannot be completely attributed to their executive function deficits.

**TABLE 5:** Differences between typically developing (TD) group and the other three groups on the response time of prospective memory after controlling for executive function.

	*B*	*SE*	*t*	*R*^2^	*F*
Step1				0.49	34.48***
N-back	0.32	0.08	3.83***		
SCWT	0.06	0.08	0.74		
MOS	0.47	0.06	5.10***		
Step2				0.55	7.87**
N-back	0.33	0.21	1.59*		
SCWT	0.01	0.13	0.04		
MOS	0.31	0.12	1.84*		
RD vs. TD	0.06	0.27	0.55		
MD vs. TD	0.15	0.22	1.71*		
RDMD vs. TD	0.06	0.26	0.57		
(RD-TD)*N-back	0.04	0.27	0.35		
(RD-TD)*SCWT	0.08	0.28	0.73		
(RD-TD)*MOS	0.04	0.23	0.38		
(MD-TD)*N-back	0.06	0.27	0.46		
(MD-TD)*SCWT	0.01	0.29	0.13		
(MD-TD)*MOS	0.13	0.24	1.29		
(RDMD-TD)*N-back	0.01	0.27	0.08		
(RDMD-TD)*SCWT	0.01	0.22	0.02		
(RDMD-TD)*MOS	0.03	0.17	0.2		

The B of “RD vs. TD” reflects the mean difference between the RD group and the TD group on the task. The B of “MD vs. TD” reflects the mean difference between the MD group and the TD group on the task. The B of “RDMD vs. TD” reflects the mean difference between the RDMD group and the TD group on the task. *p < 0.05; **p < 0.01; ***p < 0.001.

## Discussion

The present research was the first to disentangle the significance of the three main facets of executive control (shifting, inhibition, and updating) for predicting PM performance among different types of LDs. The results showed that students with MDs and RDMDs suffered from PM deficits. The deficit profile varied between groups of LDs. Students with RDMDs showed extensive deficits in PM, shifting, inhibition, and updating. In comparison, students with MDs exhibited PM and shifting deficits, while students with RDs exhibited only updating deficits. Furthermore, the results suggested that executive function deficits and PM in students with MDs were relatively independent.

### Executive Function Deficits in Students With Different Forms of Learning Disabilities

In the current study, we assessed three executive functions to investigate the executive function deficit profile for students with LDs. In terms of executive functioning skills, some previous studies confirmed that different types of LDs have different executive function deficits. The separability of the central executive function shows the specificity of students with different types of LDs in different executive function tasks. Our findings showed that students with MDs also experienced executive function problems in shifting. Students with RDs experienced updating deficits, whereas students with RDMDs suffered from extensive executive function deficits in shifting, inhibition, and updating. These findings were consistent with previous studies that have indicated impaired executive functioning in students with RDMDs (Sluis et al., 2004; Wang et al., 2008b; Peng et al., 2013; Zhang et al., 2016; Deng et al., 2018).

The results show that students with MDs (and RDMDs) had difficulties in shifting, which is consistent with previous studies (Jiao, 2014; Lee and Bull, 2016). However, no significant difference was found on inhibition and updating between the TD and MD groups. There is no reliable difference between the groups in the inhibition and updating tasks, which is not consistent with the results of Lee and Bull (2016) and Jiao and Liu (2018) suggesting that poor inhibitory control and updating was related to MD. It is possible that the criterion of the current study for designating subgroups was higher than that of Jiao (2018). Our cutoff score was at the 25th percentile, and Jiao’s study utilized a lower cut point at the 20th percentile. Therefore, we reason that with lower mathematical performance, inhibition deficits may manifest more. In the present study, the general effect of the higher cut point was to decrease the difference in the level among the students with LDs relative to typical students. Given that, the different cut points for designating subgroups may exhibit specific executive function deficits, future studies should compare results across different cut points. Doing so, may elucidate why some of these same executive function skills are weak for some students with LDs, but others are not. Moreover, we found a weak, but marginally significant difference in updating performance between MDs and TDs, and this seems to contradict the findings of Lee and Bull (2016), who reported prominent impaired updating in students in preschool or primary school with MDs. At least two explanations could account for the weak findings. First, developmental changes may explain the missing deficits of updating among students with MDs found in adolescence (present study) compared to preschool or primary school years. Second, the discrepancy in the updating task’s performance between MDs and TDs may depend on task characteristics. The results showed the floor effect in the 0-back and the ceiling effect in the 2-back task. The N-back task may not be very suitable to differentiate the updating ability in junior school students.

Furthermore, students with RDs (and RDMDs) exhibited updating deficits. This result was consistent with a previous study that indicated impaired updating in students with RDs (Cornoldi et al., 2012). Smith-Spark and Fisk’s study (Smith-Spark and Fisk, 2007) indicated that students with updating dysfunction have limited memory capacity and difficulty of performing fast and efficient operations on memory representations, and updating dysfunction may be an important cause of RDs. There was no significant difference on shifting and inhibition tasks between the TD and RD groups. However, we found that students with RDMDs had domain-general updating deficits. At first glance, our results contradict those of Hari and Renvall (2001) and Leong et al. (2007), who reported impaired inhibition and shifting in adults with RDs. However, the MD have been considered less systematically, most previous studies ignored the difference in students with MDs compared to those with RDMDs and RDs (Hari and Renvall, 2001; Leong et al., 2007; Lallier et al., 2009). This difference is important since individuals with RDs may be susceptible to both reading and mathematical difficulties (Moll et al., 2014; Cirino et al., 2015). It is possible that some students with inhibition and shifting deficits in their study were actually only reading disabled or students with RDMDs. Therefore, we cannot conclude whether these deficits are derived from pure RDs or double LDs. In the current study, we differentiated students with RDs from students with RDMDs. Thus, our findings suggest that students with RDs might not have inhibition and shifting impairments if their mathematics is intact. A key future step would be to evaluate the role of these cognitive functions in an intervention that contrasts RD, MD versus RDMD subgroups.

### Prospective Memory Deficits in Students With Different Forms of Learning Disabilities

Our results indicate that students with MDs (and RDMDs) suffered from PM deficits, which was in line with the findings of impaired event-based PM in individuals with LDs (Dong et al., 2008; Chen et al., 2017, 2018). This may be due to their deficits in the executive functions that are involved in PM (Mahy et al., 2014). The central executive is assumed to allocate resources between the maintenance of PM intentions and ongoing tasks (Chen et al., 2017). However, previous studies on the role of the three main facets of executive control (shifting, inhibition, and updating) in PM have produced an inconsistent pattern of results (Schnitzspahn et al., 2013). Mackinlay et al. (2009) found that students with higher shifting had better PM performance. Mahy and Moses (2011) used a PM task and two executive function tasks; their results indicated that updating predicted PM performance. It has even been proposed that “executive functioning” may be relevant for age-related PM performance. Then, Schnitzspahn et al. (2013) found that inhibition and shifting were strong predictors of PM performance and that they also explained the age differences in PM, although updating was not related to PM performance across adulthood. On the whole, previous results suggest that executive function predicts event-related PM. Damage to the executive functions is common in LDs, and individuals with LDs are predicted to demonstrate PM impairment stemming from impaired executive functions.

Our results showed that students with RDMDs showed extensive deficits in shifting, inhibition, and updating. Compared with the TD group, the RDMD group might have suffered from significant deficits in PM. The results of the task shifting performance tests showed that the MD group performed worse than the RD and TD groups. No significant differences were found for the inhibition and updating tasks between the MD and RD groups. Although RD students had updating deficits in executive functions compared with the TD group, the difference between the MD and RD groups was not significant. Furthermore, we found a weak, but marginally significant, difference in updating performance between the MD and TD groups. Additionally, the MD group showed PM deficits that might have been derived from an impaired shifting ability. Among the three LD groups, regarding the extent of damage in executive functions, the RDMD group was first, the MD group was second, and the RD group was last.

It should be noted that our results indicated that students with RD did not show PM deficits, which could be due to their intact shifting abilities. The preparatory attentional and memory process theory proposed that the successful performance of the prospective component of PM involves shifting between processes related to the ongoing task and processes related to evaluating responses to the environment on the periphery of one’s attentional focus (Smith, 2003; Smith and Bayen, 2004; Schnitzspahn et al., 2013). This theory especially emphasized the importance of shifting for PM, and differences in PM performance between students with MDs and RDs may be traced back to their different performances in the shifting task.

### Effect of Executive Function Deficits on Predicting PM Deficits in Students With Different Forms of Learning Disabilities

We further investigated the role of executive functioning (i.e., shifting, inhibition, and updating) on the PM deficits in different types of LDs. The results showed that PM was positively correlated with updating and shifting for each group. Participants with better updating and shifting performed better on the PM task. However, PM was not correlated with inhibitory control, which indicated that PM deficits were not affected by inhibitory control. Our results showed that most executive functions were significantly correlated with the PM tasks, confirming the notion that there is a close relation between executive functions and PM.

Stepwise regressions revealed that shifting and updating significantly predicted the PM performance for the RD group, RDMD group, and TD group. Shifting is helpful for students to flexibly change their resource allocation in the PM task and is an important guarantee for students to successfully complete the PM task. Additionally, we speculated that updating may also play an important role in PM. The better the updating is, the greater the memory span, the more room there is to store and process relevant information, and the better students perform on PM tasks. In general, many of the abilities required to successfully complete PM tasks are affected by updating (Smith and Bayen, 2004). Of course, this may also be related to the complexity of PM target clues in this study. Studies (Hicks et al., 2005) show that the complexity of the PM task may also affect updating demand. If a PM task is more complex, the updating resources needed to implement the PM are relatively large since the memory or coding PM storage space is relatively large (Mahy et al., 2014). No predictive effect of inhibitory control on the PM of LDs was found in this study which may be related to the fact that the ongoing task in this study was relatively simple for the subjects. The processing theory of PM put forward that when the ongoing task was very simple, there was no need to implement inhibition control function to monitor the PM and ongoing task (McDaniel and Einstein, 2000; Mahy and Moses, 2011). On the other hand, the PM development of MD students was significantly predicted by their shifting, which was in line with the results of Mackinlay et al. (2009).

However, after partialing out the effects of executive function, we found that there were no significant group differences between the TD and RD groups and between the TD and RDMD groups on PM tasks, and also no significant interaction effect between the group and executive functions. Only the group differences between MD group and TD group on PM tasks remained. Thus, our findings might indicate that the PM of RD and RDMD groups were mostly affected by executive function, while PM deficits of students with MDs are not completely caused by special aspects of executive function deficits, but may be other general cognitive functioning. This is consistent with the results from studies on students with autism spectrum disorder suggesting that the PM deficits in students with autism spectrum disorder were not entirely attributed to their deficits in some lower level executive function (Li et al., 2014). So, we speculated the PM deficits and executive function deficits are relatively independent for students with MDs.

In this sense, many studies had discussed the relationship between event-related PM and executive function, but the results of previous studies were inconsistent on the predictive effect of various components of executive function on PM. Therefore, future studies should complement a variety of experimental tasks to further explore the relationship between central executive function and PM.

## Limitation

The study had a number of limitations: first is the particularity of the applied cognitive tasks of executive function and PM. By using multiple indicators to evaluate each cognitive construct, the distortion caused by a single task indicator can be reduced. Future studies should use more different indicators to measure the cognitive factors of interest, and the present findings therefore await replication with other neurophysiological indicators. In addition, the sample groups consisted of students nearly from the junior school, which may also limit the generalizability of the present results to other populations. Moreover, the failure to find a correlation between the performance of the updating and inhibition tasks was surprising. Research showed that the three components of executive function accelerated from the ages of 7–12 years, but the development trend and speed of each component were different (Brocki and Bohlin, 2004). The fastest developing ability was inhibition, which had already matured by 7 years old (Klenberg et al., 2001). The ability of updating developed comparatively later than inhibition (Brocki and Bohlin, 2004). Thus, the relation between inhibition and updating in our study may be confused by each of their different critical periods.

## Conclusion

To sum up, the results suggest that executive function is an important predictor of PM in students with LDs. The current results indicate that students with MDs suffer from deficits in PM, and those with RDMDs exhibit significant impairments and weaknesses in PM, shifting, inhibition, and updating. In contrast, updating deficits were uniquely associated with RDs, whereas difficulty shifting was specifically associated with MDs deficits. Stepwise regressions showed that shifting and updating significantly predicted PM performance for the RD group, RDMD group and TD group; for the MD group, only shifting significantly predicted the PM performance, but PM deficits were not completely confined to shifting deficits. In summary, these results suggest that RDs and MDs are separate but correlated disorders that sometimes occur due to shared executive functions risk factors that lead to weaknesses in PM.

## Implications for Theoretical and Practice

These findings have important implications: theoretically, our findings support the view that learning disorders such as RDs, MDs, and RDMDs are the result of multiple executive function deficits. Furthermore, we found evidence suggesting that executive function, specifically shifting and updating, are implicated in PM. The results of the present research imply that the level of executive function influences PM in different forms of LDs. Our findings further suggest that differences in shifting ability have a larger influence on PM in students with MDs. Overall, our results underscore the importance of the three components of executive function in the study of PM in LDs. Future research needs to further examine further the more roles of executive functions in student’s PM. Generally, the essence of PM in students with LD should be more completely studied, as it is a fundamental life skill that has substantial impacts on academic and interpersonal skills.

In practice, as LDs are heterogeneous disorders, there is likely to be considerable variation in the central executive function profiles of these students. Meanwhile, it is important to assess different forms of LDs individually in all components of executive function, as this can direct the choice of interventions and can take into account the distinct profiles associated with RDs and MDs. Therefore, specific executive function training and instructions should be designed for different groups of LDs. For students with RDMDs, training tasks tapping all executive functions are recommended. Choosing inhibition tasks and updating tasks is preferred for executive function training for students with MDs. In contrast, shifting tasks and inhibition tasks are preferred for students with MDs. In other words, further investigation is needed to determine appropriate executive function training procedures for students with different forms of LDs.

## Data Availability Statement

All datasets generated for this study are included in the article/supplementary material.

## Ethics Statement

The studies involving human participants were reviewed and approved by Ethics Committee of Henan University. Written informed consent to participate in this study was provided by the participants’ legal guardian/next of kin.

## Author Contributions

LJ and YC designed the study and drafted the manuscript. XJ and LW performed the study. JZ, QZ, and HG analyzed the data and edited the manuscript. YC revised the manuscript. All the authors contributed to the article and approved the submitted version.

## Conflict of Interest

The authors declare that the research was conducted in the absence of any commercial or financial relationships that could be construed as a potential conflict of interest.
